# Rationale and design of the randomised Treatment of sleep apnoea Early After Myocardial infarction with Adaptive Servo-Ventilation trial (TEAM-ASV I)

**DOI:** 10.1186/s13063-020-4091-z

**Published:** 2020-01-31

**Authors:** Henrik Fox, Andrea Hetzenecker, Stefan Stadler, Olaf Oldenburg, Okka W. Hamer, Florian Zeman, Leonhard Bruch, Mirko Seidel, Stefan Buchner, Michael Arzt, for the TEAM-ASV I Investigators

**Affiliations:** 1 Klinik für Allgemeine und Interventionelle Kardiologie/Angiologie, Herz- und Diabeteszentrum NRW, Ruhr-Universität Bochum, Bad Oeynhausen, Germany; 2 Klinik und Poliklinik für Innere Medizin II, Universitätsklinikum Regensburg, Franz-Josef-Strauss-Allee 11, 93053 Regensburg, Germany; 3 Zentrum für Pneumologie, Klinik Donaustauf, Donaustauf, Germany; 4 Institut für Röntgendiagnostik, Universitätsklinikum Regensburg, Regensburg, Germany; 5 Zentrum für klinische Studien, Biostatistics, Universitätsklinikum Regensburg, Regensburg, Germany; 6 Klinik für Innere Medizin / Kardiologie, Unfallkrankenhaus Berlin, Berlin, Germany; 7 Innere Medizin II – Kardiologie, Sana Kliniken des Landkreises Cham, Cham, Germany

**Keywords:** Heart failure, Myocardial infarction, Sleep-disordered breathing, Myocardial salvage, Adaptive servo-ventilation, Cardiac magnetic resonance imaging

## Abstract

**Aims:**

In acute myocardial infarction (AMI), impaired myocardial salvage and large infarct size result in residual heart failure, which is one of the most important predictors of morbidity and mortality after AMI. Sleep-disordered breathing (SDB) is associated with reduced myocardial salvage index (MSI) within the first 3 months after AMI. Adaptive servo-ventilation (ASV) can effectively treat both types of SDB (central and obstructive sleep apnoea). The Treatment of sleep apnoea Early After Myocardial infarction with Adaptive Servo-Ventilation trial (TEAM-ASV I) will investigate the effects of ASV therapy, added to percutaneous coronary intervention (PCI) and optimal medical management of AMI, on myocardial salvage after AMI.

**Methods/design:**

TEAM ASV-I is a multicentre, randomised, parallel-group, open-label trial with blinded assessment of PCI outcomes. Patients with first AMI and successful PCI within 24 h after symptom onset and SDB (apnoea–hypopnoea index ≥ 15/h) will be randomised (1:1 ratio) to PCI and optimal medical therapy alone (control) or plus ASV (with stratification of randomisation by infarct location; left anterior descending (LAD) or no LAD lesion). The primary outcome is the MSI, assessed by cardiac magnetic resonance imaging. Key secondary outcomes are change of infarct size, left ventricular ejection fraction and B-type natriuretic peptide levels and disease-specific symptom burden at 12 weeks.

**Conclusion:**

TEAM ASV-I will help to determine whether treatment of SDB with ASV in the acute phase after myocardial infarction contributes to more myocardial salvage and healing.

**Trial registration:**

ClinicalTrials.gov, NCT02093377. Registered on March 21, 2014.

## Background

After acute myocardial infarction (AMI), myocardial salvage is central to limiting permanent damage to the heart because large infarct sizes result in enduring heart failure (HF) [1], which is the most important predictor of morbidity and mortality after AMI [2]. Infarct expansion is promoted by intermittent hypoxia [3, 4] and left ventricular mural pressure [1]. Both intermittent hypoxia and increased left ventricular mural pressure are hallmarks of sleep-disordered breathing (SDB) [5]. Therefore, patients with AMI may be highly sensitive to the deleterious effects of SDB [6].

Recent observational studies suggest that at least a moderate degree of SDB affects up to 66% of patients in the early phase after AMI [6, 7]. Patients with AMI are affected by two main types of SDB: obstructive sleep apnoea (OSA) and central sleep apnoea (CSA) [6, 8]. OSA is characterised by repetitive collapses of the upper airway during sleep and continued respiratory effort during apnoeas. Inspiratory efforts against the occluded pharynx during obstructive apnoeas generate exaggerated negative intrathoracic pressure that increases left ventricular transmural pressure and, as a result, afterload [5]. Additional acute consequences of OSA include repetitive oxygen desaturations, sympathetic nervous system activation, increased heart rate and elevated blood pressure [5, 9]. In contrast to OSA, there is no upper airway obstruction and no respiratory effort during central apnoeas [10]. CSA in patients with impaired cardiac function typically shows a characteristic periodic breathing pattern with a waxing and waning of tidal volume (Cheyne Stokes respiration) [10]. Similar to OSA, CSA causes intermittent nocturnal hypoxia, arousals from sleep, repetitive sympathetic nervous system activation, and swings in heart rate and blood pressure, but does not generate negative intrathoracic pressures [10].

Especially in the early phase after AMI, SDB is associated with increased cardiac workload [11], prolonged myocardial ischaemia [12], increased infarct size and reduced myocardial salvage index within the first 3 months [13]. Other negative consequences of SDB in this setting have been reported, such as worse left ventricular ejection fraction (LVEF) [14], enlargement of the right heart [15] and disturbed cardiac repolarisation [16]. All of these unfavourable aspects of SDB impair myocardial salvage after AMI, contributing to the development of HF. In addition, SDB per se promotes the development and progression of HF [10], making SDB a worthwhile target of therapy to prevent, or at least alleviate, HF development after AMI.

Previous randomised controlled trials in patients with AMI and SDB evaluated the effects of continuous positive airway pressure (CPAP) on major cardiovascular events [17, 18]. Both studies did not find a significant effect of CPAP on major cardiovascular events [17, 18]. In contrast to the present proposed study, TEAM-ASV I, cardiac magnetic resonance imaging (CMR) was not performed and myocardial salvage index was not studied [17, 18]. In a systematic review no other randomised controlled trials evaluating the effects of positive airway pressure therapy on myocardial salvage index using CMR could be identified.

Adaptive servo-ventilation (ASV) is currently the most effective therapy with respect to normalisation of nocturnal breathing in patients with impaired cardiac function plus CSA and OSA, with or without periodic breathing [19]. However, the effects of nocturnal ASV therapy on myocardial salvage, infarct mass and size and left ventricular function in patients with AMI and SDB have not been studied to date. Treatment of SDB in the early phase after AMI may constitute a new therapeutic opportunity to prevent development of HF after AMI. Considering the therapeutic equipoise of treatment of SDB in the early phase after AMI, a randomised controlled trial is justified. Therefore, the Treatment of SDB early After Myocardial infarction with Adaptive Servo-Ventilation trial (TEAM-ASV I) was designed to test the effect of ASV therapy, added to percutaneous coronary intervention (PCI) and optimal medical management, on myocardial salvage after AMI.

## Methods/design

### Study design

TEAM ASV I is a multicentre, randomised, parallel group, open-label trial with blinded assessment of outcomes. It will compare treatment with PCI and optimal medical therapy (control group) with PCI, optimal medical therapy and ASV (ASV group) in patients with AMI and SDB. The trial is an investigator-initiated and run study, funded by the ResMed Foundation (La Jolla, CA, USA); ResMed Germany (Martinsried, Germany) is providing the ASV devices. The trial was approved by the relevant institutional ethics committee (Ethikkommission der Universität Regensburg, approval number 11–101-0229) and is being conducted in accordance with the Declaration of Helsinki. All patients will provide written informed consent before undergoing study investigations. This trial has been registered with ClinicalTrials.gov (www.clinicaltrials.gov; NCT02093377).

The trial began enrolment in March 2014 and is expected to end enrolment in December 2020. As of writing, a total of 67 of the 90 target patients have been randomized.

### Study oversight

The study is monitored by an independent data safety monitoring committee (DSMC), comprising three clinical trial experts (two cardiologists and an independent statistician) who will review serious and non-serious adverse events throughout the study. An interim safety analysis is pre-specified after randomisation of the 35th patient and will include all safety variables related to this trial (study device-related and non-related events).

### Study population

Consecutive patients with their first AMI in a stable haemodynamic condition will be assessed for eligibility according to the inclusion and exclusion criteria shown in Table 1. After providing written informed consent, further evaluation of eligibility will follow, including medical history, physical examination and SDB assessment using cardiorespiratory polygraphy. The study has no gender-based selection criteria. In accordance with the epidemiology of AMI and SDB, the majority of participants (80–90%) are expected to be male [6].

**Table 1:** Inclusion and exclusion criteria

Inclusion criteria	
- Aged 18–80 years - First AMI - ST elevation in ECG or acute occlusion of coronary artery - Primarily successful PCI achieved < 24 h after onset of symptoms - SDB (apnoea-hypopnoea index ≥ 15/h of total recording time) - Written informed consent	
Exclusion criteria	
- Previous myocardial infarction - Previous myocardial revascularisation (PCI or surgical) - Indication for surgical revascularisation - Cardiogenic shock - Mean supine blood pressure < 60 mmHg - NYHA class IV - Implanted cardiac device or other contraindications for CMR - Known allergies or a contraindication to contrast dye (e.g. GFR < 30 mL/min/1.73m^2^) - History of stroke - Contraindications for positive airway pressure support (mean supine blood pressure < 60 mmHg, inability to clear secretions, risk of aspiration of gastric contents, history of pneumothorax and/or pneumomediastinum, a history of epistaxis causing pulmonary aspiration of blood) - Severe obstructive or restrictive airway disease - Heart failure due to primary valve disease - Patients on, or with indication for, oxygen therapy - Mechanical ventilation - Non-invasive ventilation - Nocturnal positive airway pressure support - Diurnal symptoms of OSA requiring immediate treatment - Awaiting heart transplantation - Pregnancy	

*AMI* acute myocardial infarction, *CMR* cardiac magnetic resonance imaging, *ECG* electrocardiogram, *GFR* glomerular filtration rate, *NYHA* New York Heart Association, *OSA* obstructive sleep apnoea, *PCI* percutaneous coronary intervention

### Randomisation

Randomisation will be performed by using the online randomization tool Randomizer (https://www.randomizer.at/) supervised by the Center of Clinical Studies. This tool ensures a secret randomization schedule for all participants and offers a 24/7 online randomization service. Randomisation to the intervention arms will be performed in a 1:1 ratio and will be stratified for infarct location (e.g. left anterior descending artery (LAD) lesion and no LAD lesion) and study site. In addition to PCI, patients randomised to the control group receive optimal medical therapy for the management of AMI according to current guidelines [20, 21]. Patients randomised to the ASV group receive optimal medical therapy for the management of AMI according to current guidelines [20, 21] plus treatment of SDB with ASV (AutoSet™ CS –Pace Wave, ResMed Corp., San Diego, CA, USA).

### Intervention: adaptive servoventilation

Following baseline assessments, patients randomised to ASV will be fitted with a comfortable nasal mask. Full face masks are only provided if the nasal mask cannot be tolerated or treatment of SDB is not possible with it (e.g. due to major mouth breathing and leakage) The patient will use the device during the daytime to achieve acclimatisation and to allow further adjustments to minimise leaks. Daytime device use will take place for 30–120 min until the patient feels comfortable with the mask and the device and leak is minimised. Night time treatment initiation will be performed under polygraphic monitoring. Default device settings include autotitrating expiratory positive airway pressure (EPAP) of 5–15 cmH_2_O with adaptive inspiratory pressure support of 0–15 cmH_2_O. Further pressure adjustments and the use of heated humidification is at the discretion of the investigator. Standardised recommendations are provided. Device usage for at least 6 h every night is recommended.

### Baseline assessments

**Fig. 1:**
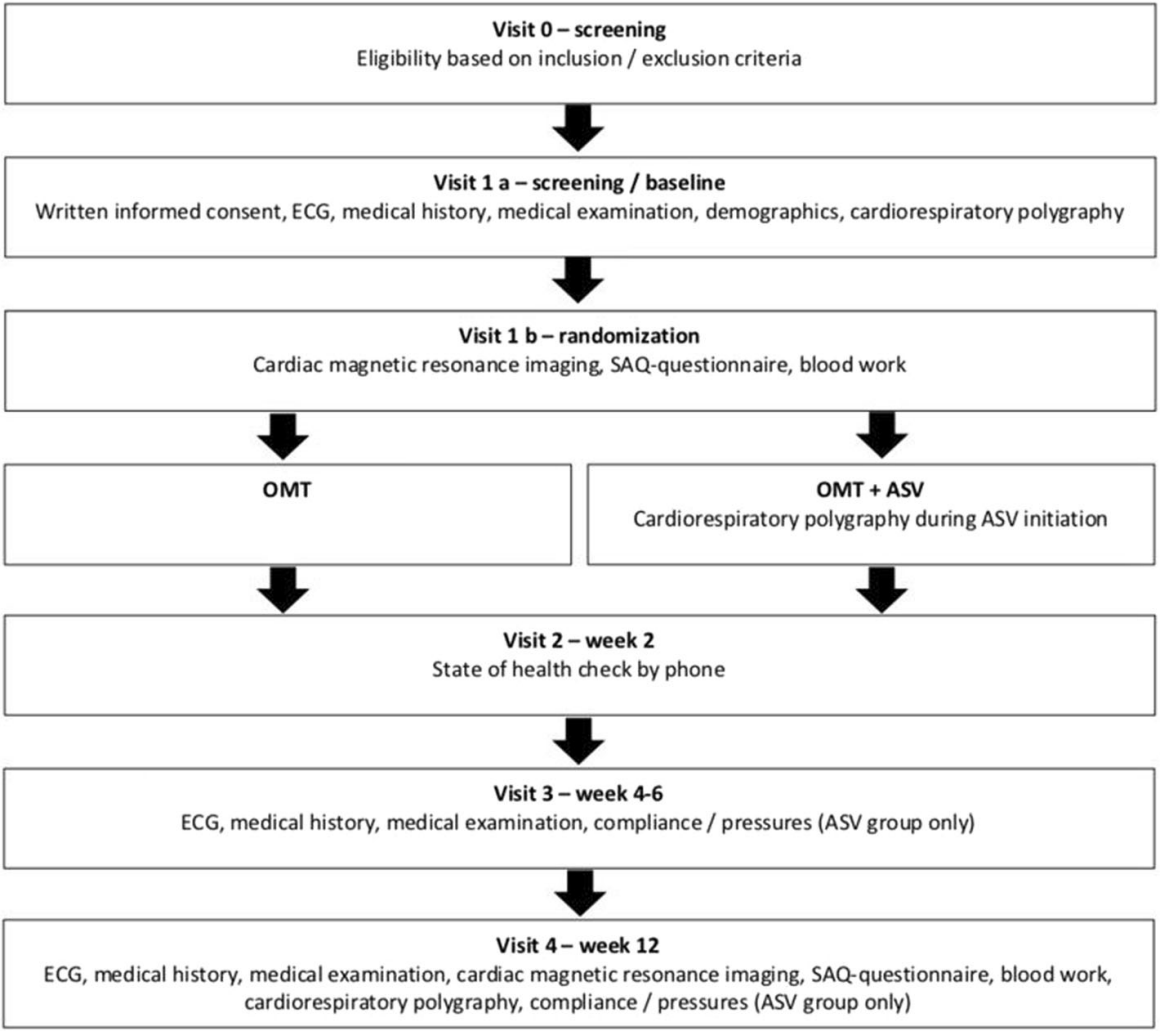
Schedule of study visits. *ASV* adaptive servo-ventilation, *ECG* electrocardiogram, *OMT* optimal medical therapy, *SAQ* Seattle Angina Questionnaire

Baseline assessments are performed in all patients meeting the inclusion and exclusion criteria. These include demographics, medical history, physical examination, ECG, cardiac magnetic resonance imaging (CMR), laboratory testing and completion of the Seattle Angina Questionnaire within 5 days after PCI (in-patient) (Fig. 1).

### Follow-up

Follow-up visits are performed after 2, 4 and 12 weeks to assess outcome measures and adverse events and to ensure compliance with the allocated treatment. Details of assessments at each visit are provided in Fig. 1. Changes of medical therapy are documented in both groups (control and ASV). In the ASV group at all follow-up visits, ASV-device data, including usage, mask leak, respiratory data and applied pressures, are collected to ensure effective therapy and compliance.

### Objectives

The primary objective is to evaluate the effects of 3 months of ASV therapy in addition to PCI and optimal medical management of AMI on myocardial salvage (myocardial salvage index (MSI)), assessed by CMR. Secondary objectives are listed in Tables 2 and 3.

**Table 2:** Secondary objectives

Secondary objectives	
To determine the effects of 12 weeks’ ASV in patients with SDB early after AMI on the following: - Infarct size - Left ventricular remodelling assessed using CMR (myocardial salvage, microvascular obstruction, change of infarct size, infarct size at 12 weeks, change in left ventricular ejection fraction, left ventricular end-systolic volume, left ventricular end-diastolic volume) - NT-proBNP levels - Disease-specific symptom burden (Seattle Angina Questionnaire) - Daytime and night time blood pressure and heart rate - Total cholesterol and low-density lipoprotein cholesterol - SDB (apnoeas and hypopnoeas per hour of sleep, mean oxygen saturation) - Renal function (GFR, calculated using the 4v-MDRD formula) - Biochemical markers of inflammation and vascular function (hsCRP, blood count, fibrinogen)	

*AMI* acute myocardial infarction, *ASV* adaptive servo-ventilation, *CMR* cardiac magnetic resonance imaging, *GFR* glomerular filtration rate, *hsCRP* high-sensitivity C reactive protein, *NT-proBNP* amino terminal-pro B-type natriuretic peptide, *SDB* sleep-disordered breathing

**Table 3:** Cardiac magnetic resonance imaging protocol

Examinations at baseline and after 12 weeks	
Infarct size: - Myocardial oedema (area at risk) - Myocardial salvage index - Left ventricular volumes and mass - Left ventricular ejection fraction	
Technical standards	
- The salvaged myocardium is defined as area at risk minus final infarct size - The myocardial salvage index (MSI) is defined as area at risk minus final infarct size as a percentage of the area at risk - The area at risk is measured 3 to 5 days after PCI and final myocardial infarct size is measured 12 weeks later - Patients will be examined in supine position with care taken to ensure identical position in the scanner for both exams - All images will be acquired using ECG gating technique - Cine images will be acquired with a steady state free precession sequence according to a systematic protocol - The entire left ventricle is covered with a stack of short axis images - Short-axis T2w-STIR imaging is used for myocardial oedema imaging - 10–15 min after bolus injection of gadolinium contrast medium (0.2 mmol/kg body weight) myocardial DE-CMR will be performed with consecutive short axis slices - All analyses will be conducted according to established standard operating procedures - Calculation of left ventricular volumes and ejection fraction is performed using standard available analysis software - The extent of myocardial oedema and delayed enhancement in each image will be quantified with standard analysis software - Regions of infarct-related area at risk (oedema) and myocardial infarction were identified as hyperintense regions within the T2w-STIR images and DE-CMR images, respectively - The myocardium at-risk region and the infarcted region is delineated automatically with manual adjustment when needed on delayed enhancement imaging - All measurements will be expressed as percentage of the total left ventricular myocardial mass and quantified in grams	

*DE-CMR* delayed enhancement cardiac magnetic resonance imaging, *ECG* electrocardiogram, *PCI* percutaneous coronary intervention, *T2w-STIR* T2-weighted short-tau inversion recovery

### Outcomes

#### Polygraphy

Polygraphy (SOMNOscreen™ plus RC, SOMNOmedics, Randersacker, Germany) will be performed within 3 days after PCI and repeated at the 12-week follow-up visit in both treatment groups (Fig. 1). Polygraphy evaluation includes breathing parameters (such as pressure cannula, snoring, thoracic and abdominal respiratory effort measures), oxygen saturation, ECG (including estimates of systolic and diastolic blood pressure based on the pulse-transit time method using validation references), recording of body position and light detection as well as a patient marker.

All polygraphy data are analysed by a central scoring lab to ensure consistency and quality. Each centre will send three respiratory recordings to the central lab for checking and validation as part of quality control. Apnoeas and hypopnoeas will be scored according to American Academy of Sleep Medicine (AASM) 2012 criteria by one experienced sleep technician blinded to the clinical data [22]. SDB is defined as an apnoea-hypopnoea index (AHI) ≥ 15/h (based on total recording time) and is categorised as predominantly OSA (≥ 50% of apnoeas obstructive) or predominantly CSA (< 50% of apnoeas obstructive).

#### Cardiac magnetic resonance imaging protocol

Myocardial salvage, infarct size and left ventricular function will be examined in both groups using CMR at baseline and after 12 weeks of treatment. The baseline CMR is performed to visualise myocardial oedema (i.e. the area at risk) and baseline infarct size. The second CMR is performed to measure the final infarct size.

All CMR images will be acquired with a 1.5 or 3.0 Tesla clinical scanner, as previously described [13]. The scan protocol will be standardised at all sites. Patients will be examined in the supine position with care taken to ensure identical position in the scanner for both exams. All images will be acquired during breath hold and using an ECG gating technique. Cine images will be acquired with a steady-state free precession sequence in two-chamber, four-chamber and short-axis planes. Short-axis T2w-STIR will be used for myocardial oedema imaging. Infarct size will be assessed in delayed enhancement short-axis images (segmented inversion recovery SSFP technique) covering the whole ventricle, acquired 10–15 min after bolus injection of gadolinium contrast (0.2 mmol/kg body weight). Image analysis for the assessment of ejection fraction, area at risk and infarct size, total left ventricular mass and volumes using a semi-automatic approach will be performed in anonymised form by blinded operators with commercially available software. The salvaged myocardium is defined as area at risk minus final infarct size. MSI (primary endpoint) is defined as area at risk minus final infarct size as a percentage of the area at risk (Fig. 1).

#### Laboratory testing

Venous blood samples (10 mL + optional 40 mL) and urine (100 mL) will be collected from every patient participating in the study during the first 2 h after morning awakening at baseline and at 12 weeks (Fig. 1). Part of each sample (10 mL) will be used to measure levels of plasma amino terminal-pro B-type natriuretic peptide (NT-proBNP), total cholesterol, low-density lipoprotein (LDL) cholesterol, serum creatinine, high-sensitivity C-reactive protein (hsCRP), blood count and fibrinogen. The examination will be done in an assigned central laboratory.

#### Disease-specific symptom burden questionnaire

Patients will be asked to complete the Seattle Angina Questionnaire [23] at baseline and at the final visit. Baseline and follow-up data will serve to document differences between these time points rather than evaluating changes over time. This approach accounts for the difficulties of quality of life assessment soon after a life-changing event (AMI). The Seattle Angina Questionnaire assesses subjective limitations of physical and daily activities by symptoms of coronary disease. Questionnaire scoring is undertaken as described previously [23].

#### Assessment and reporting of adverse events

Deaths, recurrent AMI, recurrent PCI, coronary bypass surgery, stent thrombosis, cerebral infarction, ventricular arrhythmia, rehospitalisation for heart failure or angina pectoris, cerebral infarction, syncope and other unexpected events will be documented at any visit and at first notice throughout the entire follow-up period. The investigator must report in detail all adverse signs and symptoms which are either volunteered by patients or observed during or following the course of investigational product administration. All AEs (including serious, unexpected/unanticipated device-related AEs (UADEs)) have to be documented in the CRF and source documents. Safety analyses will be performed in the safety population including participants who will have at least one post-baseline safety assessment. Patient informed consent includes long-term follow-up phone interviews after the 3-month trial period to assess mortality at 1, 3 and 5 years after randomization.

### Statistical analysis

#### Sample size calculation

The maximum possible effect of ASV therapy on MSI would be a reduction of 25, as calculated from a previous prospective observational study (MSI in no SDB versus SDB group, 77 versus 52) [13]. We consider that the minimum clinically relevant effect would be an MSI improvement of 10 in the ASV group compared with the control group. According to Eitel et al. [2] this degree of improvement in the MSI is associated with a 0.6 times lower risk of developing a major cardiovascular event after AMI. Therefore, to detect a MSI difference of 10 with a standard deviation of 15 ^13^ with a power of 1 − β = 80%, at a two-sided alpha significance level of 0.05, the required sample size for a randomised trial is 37 patients per group (total 74 patients). With an estimated loss to follow-up rate of approximately 17% based on previous observational data, 90 randomised patients (45 per group) are required.

#### Data analysis

The point estimate for the primary endpoint (MSI) will be presented as mean and standard deviation (SD) with the corresponding 95% confidence interval (CI). MSI values will be compared between the ASV and control groups by an analysis of covariance (ANCOVA) with treatment (ASV therapy and control) as the fixed factor and infarct size at baseline, infarct location and study centre as an additional covariable. The results will be presented in terms of least-squares means and corresponding 95% CI. The primary analysis will be performed on an intention-to-treat basis (ITT analysis set, all randomised patients). In addition, we will perform a sensitivity analysis in a per-protocol analysis set (PP population) to assess the robustness of the results. The PP population consists of all patients in the ITT population who show no major protocol violations. Major and minor protocol deviations will be identified before database lock. In addition, an on-treatment analysis, including patients with positive airway pressure support use for ≥ 4 h/day and an on-treatment AHI of < 10/h, will be performed. All safety data will be analysed in the safety population (all patients who had at least one post-baseline safety assessment). Additional pre-specified subgroup analyses are: LAD versus non-LAD lesions; central versus obstructive sleep apnoea; and PCI < 24 h versus ≥ 24 h after symptom onset.

No imputation methods for missing values will be used for the analyses of the primary and secondary endpoints (complete case analysis). In case of 5 to 40% missing data regarding the primary endpoint, a multiple imputation method will be used for further sensitivity analyses. If more than 40% of the data are missing, the trial results may only be considered as hypothesis generating results [24].

All secondary endpoints will be analysed in an exploratory manner and summarised using descriptive statistics. Safety variables will be analysed descriptively. The interim analysis after randomisation of the 35th patient will include safety variables only. All efficacy analyses will be performed on the ITT population and will be two-sided at a significance level of 0.05. All statistical analyses will be performed using SAS software version 9.4 or higher.

## Discussion

This randomised, controlled clinical trial addresses myocardial salvage after AMI in patients with moderate to severe SDB. MSI, the primary endpoint of the study, is the principal mechanism by which patients with AMI benefit from reperfusion therapies [20]. Available data show that CMR is a reproducible tool that identifies and quantifies myocardial salvage and correlates well with single-photon emission computed tomography and angiographic scores of myocardial salvage [25].

Ischaemia-affected myocardium, although reperfused, is at risk of necrosis when detrimental influences preventing salvage are present, such as hypoxia or myocardial wall stress [26]. In particular, hypoxia and myocardial wall stress occur during SDB, which also triggers surges in heart rate and blood pressure, as well as increased cardiac workload and afterload [27]. Furthermore, SDB has been shown to impair myocardial tissue perfusion [28], especially in lacerated myocardium after AMI [11]. Rapid revascularisation is key to increasing myocardial salvage in AMI, but SDB has been shown to be associated with higher frequencies of microvascular obstruction in the first days after AMI [12], resulting in prolonged resolution of ST deviation and greater ST elevation [12]. In a study of 223 patients, ejection fraction was found to be up to 10% lower after AMI when SDB was present, suggesting diminished MSI through SDB [14]. Similarly, observational trials in ST elevation myocardial infarction (STEMI) patients found impaired systolic retrograde coronary flow, worse LVEF, more regional wall motion abnormalities (more infarcted area) and less ST-segment resolution when SDB was present [28, 29]. In addition, higher peak troponin level and a longer length of stay in the coronary care unit have been linked to OSA in STEMI patients [30].

Myocardial salvage predicts mortality and major adverse cardiovascular event (MACE) outcomes in acute reperfused STEMI, independent of other known risk factors [2]. In one study, the hazard ratio for myocardial salvage as a predictor of a MACE within a 6-month follow-up period was 0.95 (95% CI 0.93–0.97) [2] and in previous observational studies, patients with reperfused AMI without SDB had a significantly greater MSI versus those with SDB (77 vs 52), showing that MSI is a stronger indicator of MACE and mortality than infarct size [13]. Therefore, myocardial salvage is an appropriate, reproducible and clinically important endpoint in clinical trials to test the efficacy of reperfusion therapy or other strategies to prevent further expansion of myocardial infarction in the early phase after reperfusion [2].

In this regard, ASV treatment of SDB may alleviate the deleterious effects of SDB after AMI and increase myocardial salvage. This is likely to occur due to the documented beneficial effects of ASV on heart rate, heart rhythm, blood pressure and systolic left ventricular transmural pressure [5, 31]. Treatment of SDB also has the potential to normalise nitric oxide-mediated vasodilation [32], potentially contributing to improved myocardial perfusion, and may support healing of stunned myocardium after AMI. Data from an observational study demonstrated lower rates of recurrent AMI and revascularisation when OSA was treated [33].

It is important to determine whether ASV therapy has beneficial effects on myocardial salvage in patients with AMI and SDB. If this is the case, ASV would be a novel concept for AMI treatment. Additional secondary endpoints have been incorporated into this trial to determine the effects of ASV therapy on myocardial function and heart failure parameters, with the goal of determining whether increasing myocardial salvage might contribute to reducing, or even preventing, the development of heart failure after AMI.

In conclusion, TEAM-ASV-I is designed to investigate the hypothesis that ASV treatment can improve myocardial salvage after AMI in patients with SDB in a randomised controlled setting, with the goal of improving our understanding of the management of SDB in patients after AMI and its effects on myocardial healing.

### Highlights of this study

- Randomized controlled, multicentre trial.
- Hypothesis is to increase myocardial salvage in patients with acute myocardial infarction and sleep-disordered breathing with adaptive servoventilation.
- Targeting and treating highly common comorbidity sleep-disordered breathing.
- Sleep-disordered breathing is known to significantly impair myocardial salvage.
- Myocardial salvage is quantified by cardiac magnetic resonance imaging.

### Trial status

This trial is still recruiting patients, protocol version 6.0, March 21st, 2014. Recruitment began after trial registration on March 21st, 2014 and enrolment is estimated to end December 2020.

## Supplementary information

**Additional file 1:** SPIRIT 2013 Checklist: Recommended items to address in a clinical trial protocol and related documents*

## Data Availability

Not applicable. This manuscript does not contain any data, as this is a study design manuscript.
