# Supplementary material for: Rationale and design of the randomised Treatment of sleep apnoea Early After Myocardial infarction with Adaptive Servo-Ventilation trial (TEAM-ASV I)
Source: Trials. 2020 Jan 31;21:129. doi: 10.1186/s13063-020-4091-z (PMC6995094; doi:10.1186/s13063-020-4091-z)
Supplement: Supplementary file 1 — Additional file 1: SPIRIT 2013 Checklist: Recommended items to address in a clinical trial protocol and related documents* [file 13063_2020_4091_MOESM1_ESM.docx]

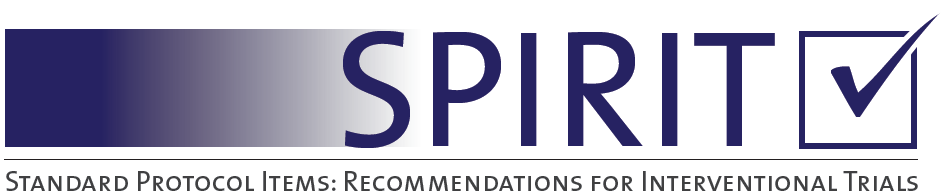


SPIRIT 2013 Checklist: Recommended items to address in a clinical trial protocol and related documents*

| Section/item | ItemNo | Description |
| --- | --- | --- |
| **Administrative information** | | |
| Title  This information is provided on page 1 in this manuscript | 1 | Descriptive title identifying the study design, population, interventions, and, if applicable, trial acronym |
| Trial registration  This information is provided on page 2 in this manuscript | 2a | Trial identifier and registry name. If not yet registered, name of intended registry |
|  | 2b | All items from the World Health Organization Trial Registration Data Set |
| Protocol version  This information is provided on page 16 in this manuscript | 3 | Date and version identifier |
| Funding  This information is provided on page 14 in this manuscript | 4 | Sources and types of financial, material, and other support |
| Roles and responsibilities  This information is provided on page 15 in this manuscript | 5a | Names, affiliations, and roles of protocol contributors |
|  | 5b | Name and contact information for the trial sponsor |
|  | 5c | Role of study sponsor and funders, if any, in study design; collection, management, analysis, and interpretation of data; writing of the report; and the decision to submit the report for publication, including whether they will have ultimate authority over any of these activities |
|  | 5d | Composition, roles, and responsibilities of the coordinating centre, steering committee, endpoint adjudication committee, data management team, and other individuals or groups overseeing the trial, if applicable (see Item 21a for data monitoring committee) |

| Introduction |  |  |
| --- | --- | --- |
| Background and rationale  This information is provided on page 4 in this manuscript | 6a | Description of research question and justification for undertaking the trial, including summary of relevant studies (published and unpublished) examining benefits and harms for each intervention |
|  | 6b | Explanation for choice of comparators |
| Objectives  This information is provided on page 8 in this manuscript | 7 | Specific objectives or hypotheses |
| Trial design  This information is provided on page 5 in this manuscript | 8 | Description of trial design including type of trial (eg, parallel group, crossover, factorial, single group), allocation ratio, and framework (eg, superiority, equivalence, noninferiority, exploratory) |
| Methods: Participants, interventions, and outcomes | | |
| Study setting  This information is provided on page 5 in this manuscript | 9 | Description of study settings (eg, community clinic, academic hospital) and list of countries where data will be collected. Reference to where list of study sites can be obtained |
| Eligibility criteria  This information is provided on page 21 in this manuscript | 10 | Inclusion and exclusion criteria for participants. If applicable, eligibility criteria for study centres and individuals who will perform the interventions (eg, surgeons, psychotherapists) |
| Interventions  This information is provided on page 6 in this manuscript | 11a | Interventions for each group with sufficient detail to allow replication, including how and when they will be administered |
|  | 11b | Criteria for discontinuing or modifying allocated interventions for a given trial participant (eg, drug dose change in response to harms, participant request, or improving/worsening disease) |
|  | 11c | Strategies to improve adherence to intervention protocols, and any procedures for monitoring adherence (eg, drug tablet return, laboratory tests) |
|  | 11d | Relevant concomitant care and interventions that are permitted or prohibited during the trial |
| Outcomes  This information is provided on page 8 in this manuscript | 12 | Primary, secondary, and other outcomes, including the specific measurement variable (eg, systolic blood pressure), analysis metric (eg, change from baseline, final value, time to event), method of aggregation (eg, median, proportion), and time point for each outcome. Explanation of the clinical relevance of chosen efficacy and harm outcomes is strongly recommended |
| Participant timeline  This information is provided on page 26 in this manuscript | 13 | Time schedule of enrolment, interventions (including any run-ins and washouts), assessments, and visits for participants. A schematic diagram is highly recommended (see Figure) |
| Sample size  This information is provided on page 10 in this manuscript | 14 | Estimated number of participants needed to achieve study objectives and how it was determined, including clinical and statistical assumptions supporting any sample size calculations |
| Recruitment  This information is provided on page 10 in this manuscript | 15 | Strategies for achieving adequate participant enrolment to reach target sample size |
| **Methods: Assignment of interventions (for controlled trials)** | | |
| Allocation: |  |  |
| Sequence generation  This information is provided on page 6 in this manuscript | 16a | Method of generating the allocation sequence (eg, computer-generated random numbers), and list of any factors for stratification. To reduce predictability of a random sequence, details of any planned restriction (eg, blocking) should be provided in a separate document that is unavailable to those who enrol participants or assign interventions |
| Allocation concealment mechanism  This information is provided on page 6 in this manuscript | 16b | Mechanism of implementing the allocation sequence (eg, central telephone; sequentially numbered, opaque, sealed envelopes), describing any steps to conceal the sequence until interventions are assigned |
| Implementation  This information is provided on page 6 in this manuscript | 16c | Who will generate the allocation sequence, who will enrol participants, and who will assign participants to interventions |
| Blinding (masking)  This information is provided on page 5 in this manuscript | 17a | Who will be blinded after assignment to interventions (eg, trial participants, care providers, outcome assessors, data analysts), and how |
|  | 17b | If blinded, circumstances under which unblinding is permissible, and procedure for revealing a participant’s allocated intervention during the trial |

| **Methods: Data collection, management, and analysis** | | |
| --- | --- | --- |
| Data collection methods  This information is provided on page 10 in this manuscript | 18a | Plans for assessment and collection of outcome, baseline, and other trial data, including any related processes to promote data quality (eg, duplicate measurements, training of assessors) and a description of study instruments (eg, questionnaires, laboratory tests) along with their reliability and validity, if known. Reference to where data collection forms can be found, if not in the protocol |
|  | 18b | Plans to promote participant retention and complete follow-up, including list of any outcome data to be collected for participants who discontinue or deviate from intervention protocols |
| Data management  This information is provided on page 10 in this manuscript | 19 | Plans for data entry, coding, security, and storage, including any related processes to promote data quality (eg, double data entry; range checks for data values). Reference to where details of data management procedures can be found, if not in the protocol |
| Statistical methods  This information is provided on page 10 in this manuscript | 20a | Statistical methods for analysing primary and secondary outcomes. Reference to where other details of the statistical analysis plan can be found, if not in the protocol |
|  | 20b | Methods for any additional analyses (eg, subgroup and adjusted analyses) |
|  | 20c | Definition of analysis population relating to protocol non-adherence (eg, as randomised analysis), and any statistical methods to handle missing data (eg, multiple imputation) |
| **Methods: Monitoring** | | |
| Data monitoring  This information is provided on page 6 in this manuscript | 21a | Composition of data monitoring committee (DMC); summary of its role and reporting structure; statement of whether it is independent from the sponsor and competing interests; and reference to where further details about its charter can be found, if not in the protocol. Alternatively, an explanation of why a DMC is not needed |
|  | 21b | Description of any interim analyses and stopping guidelines, including who will have access to these interim results and make the final decision to terminate the trial |
| Harms  This information is provided on page 6 in this manuscript | 22 | Plans for collecting, assessing, reporting, and managing solicited and spontaneously reported adverse events and other unintended effects of trial interventions or trial conduct |
| Auditing  This information is provided on page 6 in this manuscript | 23 | Frequency and procedures for auditing trial conduct, if any, and whether the process will be independent from investigators and the sponsor |

| Ethics and dissemination | | |
| --- | --- | --- |
| Research ethics approval  This information is provided on page 5 in this manuscript | 24 | Plans for seeking research ethics committee/institutional review board (REC/IRB) approval |
| Protocol amendments  This information is provided on page 5 in this manuscript | 25 | Plans for communicating important protocol modifications (eg, changes to eligibility criteria, outcomes, analyses) to relevant parties (eg, investigators, REC/IRBs, trial participants, trial registries, journals, regulators) |
| Consent or assent  This information is provided on page 5 in this manuscript | 26a | Who will obtain informed consent or assent from potential trial participants or authorised surrogates, and how (see Item 32) |
|  | 26b | Additional consent provisions for collection and use of participant data and biological specimens in ancillary studies, if applicable |
| Confidentiality  This information is provided on page 5 in this manuscript | 27 | How personal information about potential and enrolled participants will be collected, shared, and maintained in order to protect confidentiality before, during, and after the trial |
| Declaration of interests  This information is provided on page 14 in this manuscript | 28 | Financial and other competing interests for principal investigators for the overall trial and each study site |
| Access to data  This information is provided on page 5 in this manuscript | 29 | Statement of who will have access to the final trial dataset, and disclosure of contractual agreements that limit such access for investigators |
| Ancillary and post-trial care  This information is provided on page 5 in this manuscript | 30 | Provisions, if any, for ancillary and post-trial care, and for compensation to those who suffer harm from trial participation |
| Dissemination policy  This information is provided on page 5 in this manuscript | 31a | Plans for investigators and sponsor to communicate trial results to participants, healthcare professionals, the public, and other relevant groups (eg, via publication, reporting in results databases, or other data sharing arrangements), including any publication restrictions |
|  | 31b | Authorship eligibility guidelines and any intended use of professional writers |
|  | 31c | Plans, if any, for granting public access to the full protocol, participant-level dataset, and statistical code |
| Appendices |  |  |
| Informed consent materials  This information is provided on page 5 in this manuscript | 32 | Model consent form and other related documentation given to participants and authorised surrogates |
| Biological specimens  This information is provided on page 9 in this manuscript | 33 | Plans for collection, laboratory evaluation, and storage of biological specimens for genetic or molecular analysis in the current trial and for future use in ancillary studies, if applicable |

*It is strongly recommended that this checklist be read in conjunction with the SPIRIT 2013 Explanation & Elaboration for important clarification on the items. Amendments to the protocol should be tracked and dated. The SPIRIT checklist is copyrighted by the SPIRIT Group under the Creative Commons “Attribution-NonCommercial-NoDerivs 3.0 Unported” license.

TEAM-ASV I trial: Schedule of enrolment, interventions and assessments

(SPIRIT figure)

|  | **STUDY PERIOD** | | | | |
| --- | --- | --- | --- | --- | --- |
|  | **Enrolment** | **Allocation** | **Post-allocation** | | **Close-out** |
| **TIMEPOINT** | ***-5days^†^*** | **0** | ***2 weeks*** | ***4-6 weeks*** | ***12 weeks*** |
| **Visit No.** | Visit 1a | Visit 1b | Visit 2 | Visit 3 | Visit 4 |
| **ENROLMENT:** |  |  |  |  |  |
| **Eligibility screen** | X |  |  |  |  |
| **Informed consent** | X |  |  |  |  |
| **Demographics and medical history** | X |  |  |  |  |
| **Allocation** |  | X |  |  |  |
| **INTERVENTIONS:** |  |  |  |  |  |
| ***TREATMENT: ASV therapy plus guideline conform cardiac therapy**** |  |  |  |  |  |
| ***CONROL: Guideline conform cardiac therapy**** |  |  |  |  |  |
| **ASSESSMENTS:** |  |  |  |  |  |
| **Electrocardiogram, medical examination, vital signs** | X |  |  | X | X |
| ***CMR*** |  | X |  |  | X |
| ***SAP Questionnaire*** |  | X |  |  | X |
| ***Blood work***** |  | X |  |  | X |
| ***PG (1) ≤3 days after PCI*** | X |  |  |  |  |
| ***PG (2) ASV-initiation*** |  | X |  |  |  |
| ***PG (2a) on ASV-initiation (optional)*** |  | X |  |  |  |
| ***Phone interview*** |  |  | X |  |  |
| ***PG (3) (ASV/Control)*** |  |  |  |  | X |
| ***Adverse Events*** |  |  |  |  |  |
| ***Compliance of interventions*** |  |  | X | X | X |

**Definitions of abbreviations:** ECG – electrocardiogram; PG – polygraphy; PCI – percutaneous coronary intervention; ASV – adaptive servoventilation; CMR - Cardiac Magnetic Resonance Imaging; SAP-Questionnaire - Seattle Angina Pectoris Questionnaire; NT-proBNP - N-terminal pro brain natriuretic peptide; hsCRP – high sensitivity C-reactive Protein

***^†^***Assessment should be performed within the first 5 days after primary coronary intervention, when intensified monitoring on intensive care unit is not required any more (Creatine kinase <500 U/l).

Patients, who are screen failure after PG1 or day-time demonstration ASV, do not proceed to Visit1b.

*optimal medical therapy for the management of myocardial infarction according to the current guidelines of the European Society of Cardiology

** *cre*
